# Limb fracture, embolization, and penetration of the heart: a case report of a complication of unretrieved inferior vena cava filters

**DOI:** 10.1007/s12055-025-02026-2

**Published:** 2025-08-26

**Authors:** Louis Fernandez Chai, Derek Kwasi Afflu, Akshay Chauhan, Hiromu Kehara, Mikiko Senzai, Yoshiya Toyoda

**Affiliations:** Division of Cardiac Surgery, Department of Surgery, Temple University Hospital, 3401 N. Broad Street, Suite 501-C, Philadelphia, 19140 PA USA

**Keywords:** IVC filter, Strut fracture, Embolization, Foreign body, Cardiac injury

## Abstract

Inferior vena cava filters are commonly used in patients with venous thromboembolism who cannot tolerate anticoagulation. While retrievable filters are preferred, many remain in place long-term, increasing the risk of complications such as filter strut fracture and embolization. We present a case of a 47-year-old female with an incidentally discovered intracardiac filter strut fragment, 16 years post-implantation. Minimally invasive surgical removal was performed, while an additional embolized fragment in the pulmonary artery was managed conservatively. This case underscores the importance of timely filter retrieval to prevent complications and highlights management considerations for embolized struts.

## Introduction

Common indications for inferior vena cava filter (IVCF) placement include patients with venous thromboembolisms (VTE) who cannot tolerate anticoagulation, as adjuncts to anticoagulation in patients with recurrent VTE, concurrent pulmonary embolisms (PE), and deep vein thrombosis (DVT), or during lower extremity thrombolysis procedures [1]. IVCFs may be permanent or retrievable, with the latter being more common in practice.

Despite recommendations for retrieval once PE risk is abated, about two-thirds of filters are not retrieved [2]. While most remain asymptomatic, complications may develop such as occlusion from clot burden, migration, or fracture. Patients may be asymptomatic or present with symptoms associated with the location of the embolized fragment. In cases involving the cardiopulmonary system, chest pain, shortness of breath, or arrhythmias may develop, and the resulting injuries may require intervention. We present a case of a 16-year-old IVCF with two embolized struts. We describe the workup, management algorithm employed, and outcome.

## Case report

**Fig. 1:**
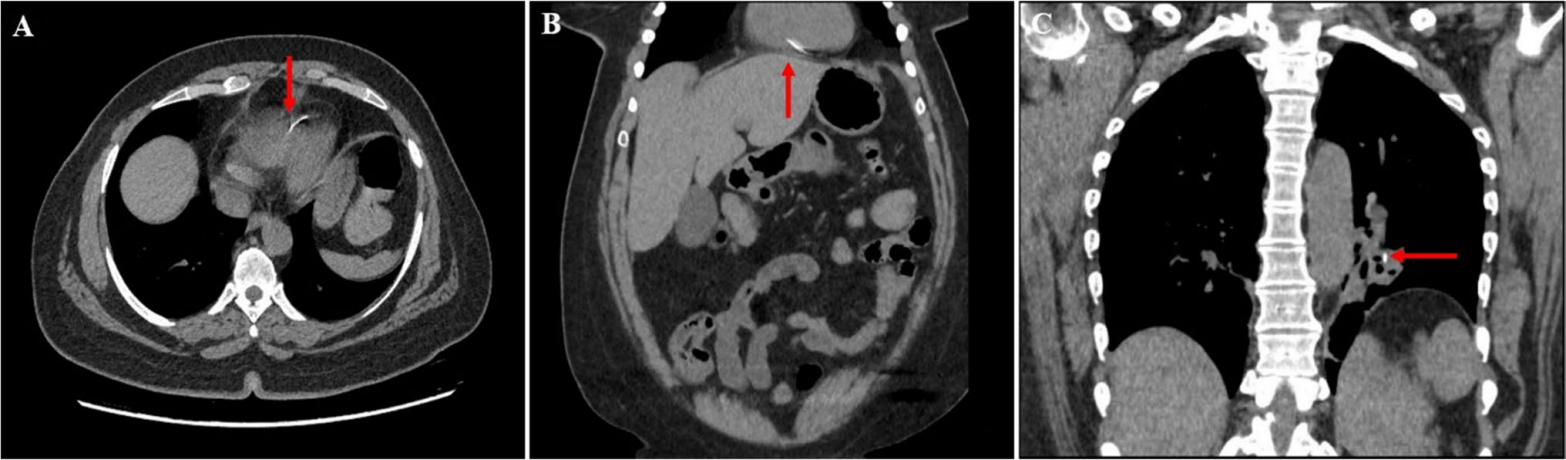
**A**, **B** Axial and coronal reconstruction of computed tomography (CT) showing linear density noted in the right ventricle. **C** Coronal CT-chest demonstrating inferior vena cava filter strut embolized to left inferior pulmonary artery segmental branch

A 47-year-old female presented with bloody diarrhea for which she obtained a computed tomography (CT) scan of the chest, abdomen, and pelvis (Fig. 1a, b).

**Fig. 2:**
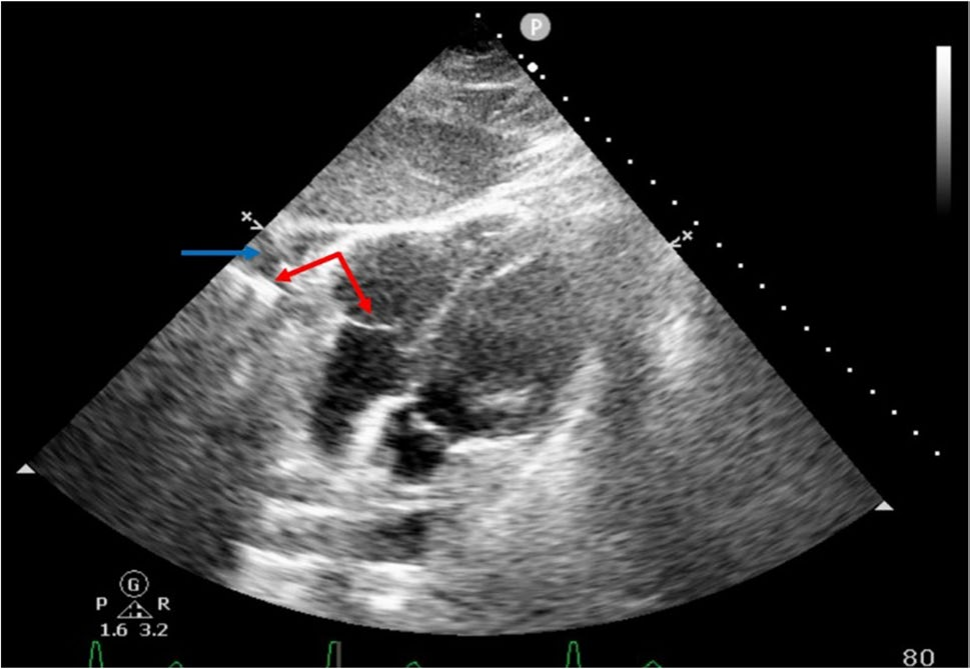
Transthoracic echocardiogram demonstrating an inferior vena cava filter strut traversing the right ventricular free wall (red arrow). This is accompanied by pericardial effusion (blue arrow)

An incidental linear metallic density was identified puncturing the right ventricle (RV). A transthoracic echocardiogram (TTE) obtained showed trace pericardial effusion and highlighted the embolized strut traversing the RV free wall (Fig. 2).

Further history revealed a Bard G2 Express IVCF prophylactically placed 16 years prior following several lower extremity orthopedic interventions. Further imaging review identified only 10 of the 12 expected struts on the intact IVCF. One of the fractured struts was the density puncturing the RV and the other was identified in a branch of the left pulmonary artery (PA) without evidence of bleeding (Fig. 1c). The patient was asymptomatic, but given the concern for RV injury, the patient was taken to the operating room for strut removal and repair.

**Fig. 3:**
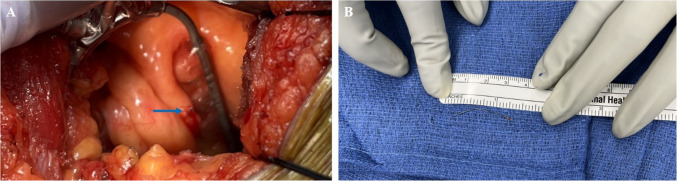
**A** Inferior vena cava filter strut protruding through the right ventricular free wall (blue arrow). **B** A 3-cm inferior vena cava filter strut following removal from the heart

Given the location near the apex, a minimally invasive subxiphoid approach was chosen. When the pericardial cavity was entered, there was trace serosanguinous effusion. The strut was not visible, but palpable near the apex. Using a stabilizer, the epicardium was incised and the strut was identified, puncturing through the RV wall (Fig. 3).

This was removed, the hole repaired primarily, and the pericardial window was formalized. Given the location of the 12th strut, the decision was made not to explore and remove it based on previously reported cases [3]. Post-operatively, the patient recovered well and was maintained on therapeutic anticoagulation. The IVCF was removed during the same hospitalization, and the remaining embolized strut will be monitored with serial imaging.

## Comment

Unretrieved IVCFs are common despite the predominance of retrievable filters being placed. There is an incremental rise in all filter-related complications with longer indwelling times, but particularly with rates of limb or strut fracture. It is hypothesized that prolonged placement results in mechanical strain and structural fatigue from the exposure to systemic blood flow or increased clot burden, leading to the fracture [4]. Fracture rates have a linear relationship with the duration of placement and can be up to 50% after 4 years [4].

Diagnosis of a fractured, embolized IVCF limb is often incidental. The radiopaque struts can easily be missed on x-rays due to the small size or obscuring overlying tissue. Additionally, radiopaque linear densities are not pathognomonic for embolized IVCF struts and can be attributed to surgical clips or superimposed external objects. Cross-sectional imaging may better identify intra-corporeal foreign bodies but still requires a high index of suspicion. A thorough history to confirm previous IVCF placement and specific brand is critical to determine duration and technical details, such as limb numbers associated with specific IVCFs, which can be used to determine the number of embolized struts. Correctly identifying the number of emboli and their locations guides management and confirms intact IVCF retrieval once externalized.

Fractured IVCF limbs most commonly remain local to the original placement site and may cause inferior vena cava (IVC) perforation, which has been reported as up to 20% of all complications [1, 5]. However, distal migration and embolization may occur, and struts have been identified in the lungs, heart, liver, kidneys, and intestines [6, 7]. Limb fractures may be asymptomatic even with distal migration but can perforate viscera or cause significant bleeding [7]. Of particular concern is intracardiac limb embolization. Though this may also be clinically silent, chest pain, shortness of breath, syncope, arrhythmias, tamponade, and even sudden death may occur [7, 8].

There is no consensus on management of embolized IVCF struts. Previous reports have advocated for no intervention with or without close surveillance for asymptomatic patients with the prevailing theory that the risk of intervention was greater than observation for an embolus that may have been present for a long duration prior to incidental discovery [3]. This is reported to be the case for patients with fractured limbs in the pulmonary vasculature. Others have advocated for systemic anticoagulation, though this is not universal. We opted to observe our patient in line with current literature for the embolized strut in the pulmonary artery. However, for intracardiac strut embolization, there is a stronger case for intervention [9]. While some reports highlight asymptomatic patients, there is a much higher incidence of life-threatening complications with intracardiac limb fracture embolization. Structural damage, arrhythmogenic irritation, and myocardial injury have all been described and are indications for emergent intervention. Given the potential for life-threatening consequences, endovascular or open surgical retrieval should be considered, even for asymptomatic and incidentally discovered intracardiac strut emboli. Exceptions to this may be patients who are poor operative candidates. Our patient was otherwise healthy and we retrieved the strut minimally invasively rather than via a full sternotomy. Additional intervention should include IVCF removal as the longer the IVCF remains, the greater the risk of further complications. On retrieval, the IVCF should be examined to corroborate imaging findings of limb number as missing struts may indicate further fractures that occurred during retrieval and require additional intervention.

## Conclusion

IVCFs are used for a broad range of indications. While uncommon, IVCF fracture and embolization can have severe consequences, and improvement in retrieval rates can help reduce morbidities. For patients presenting with cardiopulmonary strut emboli, management should be based on location and symptoms, along with IVCF retrieval.

## Data Availability

The data that support the findings of this study are available on request from the corresponding author in a deidentified format. The data are not publicly available due to Health Insurance Portability and Accountability Act (HIPAA) restrictions.
